# Experience with Tetanus in a Tertiary Care Hospital in Sudan: A Retrospective Review

**DOI:** 10.1155/2021/4818312

**Published:** 2021-12-21

**Authors:** Mumen Abdalazim Dafallah, Esraa Ahmed Ragab, Omer Ali Mohamed Ahmed Elawad

**Affiliations:** Faculty of Medicine, University of Gezira, Wad Medani, Gezira State, Sudan

## Abstract

**Introduction:**

Tetanus is still a major health issue, especially in rural areas, and is associated with high morbidity and mortality rate. This study was conducted to describe the pattern of presentation and treatment outcome among adult patients infected with tetanus in our environment.

**Materials and Methods:**

This is a descriptive retrospective hospital-based study conducted in Wad Medani teaching hospital, central Sudan. A total of thirty-one patients were enrolled in this study in the period between January 2018 and December 2020.

**Results:**

Thirty-one patients were infected with tetanus during the study period. They were 23 (74.2%) males and 8 (25.8%) females with a male-to-female ratio of 2.875 : 1. Their ages ranged from 20 to 70 years, and most of them (48.4%) were free workers. Acute injuries were the most common portal of entry (64.51%), and commonly involved the lower limbs (48.38%). Lock jaw (54.8%), muscle spasm (51.6%), and neck pain and stiffness (45.2%) were the most common presentation. Supportive measures along with surgical toilet and debridement, human tetanus immunoglobulin, antibiotics, and muscle relaxants were initiated in all patients. The most common antibiotics used were Penicillin V and Ceftriaxone. A muscle relaxant was administered to aid in relieving the spasms. Complication rate was 61.29% and included pulmonary and cardiovascular complications. Fifteen patients died accounting for an overall mortality rate of 48.4%.

**Conclusions:**

Tetanus remains a disease with high morbidity and mortality. The unknown/incomplete vaccination status among study participants, inadequate management, and lack of equipped resources lead to a devastating outcome as in Sudan.

## 1. Introduction 

The World Health Organization (WHO) defined tetanus as an acute infection caused by a bacterium *Clostridium tetani* that generates a neurotoxin [1]. *Clostridium tetani* is a Gram-positive, spore-forming, obligate anaerobic bacillus that is found in soil, dust, or animal feces. Tetanus is a preventable illness; this statement is highlighted by the WHO reporting an improvement in the mortality rate following vaccination [2]. Despite the vaccination program, tetanus is still a major issue around the world, especially in developing countries where the mortality rate is 20–45% [2, 3]. This high incidence is due to poor wound care and lack of awareness regarding immunization in developing countries [4]. The incidence and mortality rate from tetanus are lower in developed countries; this is due to early treatment and the availability of equipped intensive care units that are not found in many resource-limited hospitals in developing countries [2].

Tetanus infection usually occurs following injury, surgery, burn, ulcer, or gangrene. Snake and dog bites are also documented [3]. The bacteria generate a powerful neurotoxin (tetanospasmin), which is responsible for the clinical manifestations of tetanus. Lock jaw, generalized muscle spasms, neck stiffness, and dysphagia are the most common presenting symptoms [5].

The diagnosis is made primarily on clinical manifestations; no laboratory or imaging tests are needed to establish the diagnosis [6]. Treatment varies and depends on the severity of the disease; however, the initiation of early supportive measures, antibiotics administration, muscle relaxant, and tetanus immunoglobulin with early and effective wound debridement are keys to control the disease and preventing complications [7, 8].

In Sudan, tetanus is still a major health issue, especially in rural areas, and is associated with a high morbidity and mortality rate. There is a lack of data on the pattern and outcome of tetanus among adults in Sudan. This study aims to describe the pattern of presentation and treatment outcome among adult patients infected with tetanus in our environment and to identify the commonest causative factors.

## 2. Materials and Methods

### 2.1. Study Design

The study was a retrospective descriptive hospital-based study.

### 2.2. Study Area

The study was conducted in Wad Medani teaching hospital, central Sudan. It is a tertiary hospital serving the Gezira state and the nearby states. The hospital has an isolation room for patients infected with tetanus, and it received patients from the Gezira state and the nearby states.

### 2.3. Study Duration

The study was conducted between January 2018 and December 2020.

### 2.4. Study Population

All patients infected with tetanus and admitted to Wad Medani teaching hospital.

The diagnosis of tetanus was entirely clinical and based on the presence of one or more of the following:

- Rigidity of the neck and/or abdomen/neck stiffness
- Lock jaw
- Muscle spasm

### 2.5. Inclusion Criteria of the Study Population

The inclusion criteria included patients above 18 years, patients of both genders, and patients infected with tetanus during the period of the study.

### 2.6. Exclusion Criteria of the Study Population

The exclusion criteria included patients less than 18 years (the hospital does not accept/treat pediatric patients who are less than 18 years).

### 2.7. Sampling Technique

The sampling technique was full coverage of all patients infected with tetanus who were admitted to Wad Medani teaching hospital during the study period.

### 2.8. Data Collection Tools and Methods

The data were collected from the patients records using a questionnaire. The questionnaire contains the following data: age, gender, occupation, etiology, incubation period, comorbidities, site of the injury, presenting symptoms, types of antibiotics and muscle relaxant used, whether mechanical ventilation was used, length of hospital stay, complications, and the outcome. Prior immunization was recorded in all study participants.

### 2.9. Independent and Dependent Variables

Independent variables included the following:

- Demographic data―age and gender
- Occupation
- The causative factor

- Complications
- Patient outcome
- Length of hospitalization

### 2.10. Data Analysis

Data were analyzed using the Statistical Package for Social Sciences (SPSS 23.0) and Microsoft Excel. Descriptive statistics were applied to describe the pattern of the data. The *P* value will be considered significant if it is less than 0.05.

## 3. Results

**Figure 1:**
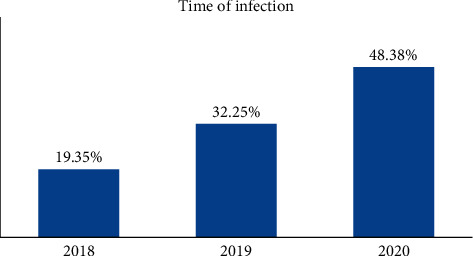
The distribution of time of infection through years (*N* = 31).

**Figure 2:**
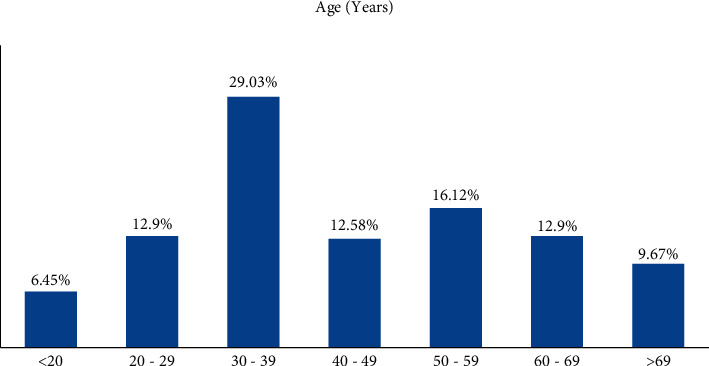
The age distribution among study participants (*N* = 31).

Thirty-one patients were infected with tetanus during the study period. The majority, 15 (48.38%), were infected in 2020 (Figure 1). These included 23 (74.2%) males and 8 (25.8%) females with a male-to-female ratio of 2.875 : 1. Their ages ranged from 20 to 70 years (Figure 2). The patients' occupation includes free worker (48.4%), farmer (19.4%), civil servant (3.2%), housewife (22.6%), student (3.2%), and retired (3.2%) (Table 1). Three patients refused to participate in the study.

Only seven (22.58%) patients reported previous tetanus immunization, while the other twenty-four (77.42%) patients were not vaccinated or did not know their tetanus immunization status.

Acute injuries such as puncture, prick, or laceration were the commonest portal of entry point (64.51%), followed by snake bites (9.67%); scorpion sting and local surgical procedures accounted for 3.22% each. The portals of entry were not identified in 19.35% of cases. Most of the cases were in the lower extremities (48.38%), and in 19.35%, the site of injury was not identified. Only 3 patients had comorbidities; i.e., 2 patients had diabetes mellitus and 1 patient had hypertension. None of the patients received the DTP, Td, Tdp, or Tdap vaccines (Table 2).

The time between the inoculation of the wound and the onset of the symptoms is known as the incubation period, and it was known in 80.65% of patients. The incubation period ranged from 2 to 25 days with a mean of 9 days and a median of 7 days. The median of the incubation period was 7 days with an interquartile range of 11 days. When the patients presented to the emergency department, the presenting symptoms were different: lock jaw was the most common presentation accounting for 54.8%, followed by muscle spasm (51.6%), neck pain and stiffness (45.2%), convulsions (38.7%), and back pain (25.8%) (Table 2).

All patients were admitted to the isolation room in the general wards; the intensive care unit was not equipped to receive patients infected with tetanus and was not supported with mechanical ventilation, and so none of the patients was admitted there. Upon admission to the hospital, supportive treatment such as fluids, prevention of gastric stress ulcer, and prevention of pressure sores was provided to all patients. Positive treatment including surgical toilet and debridement, human tetanus immunoglobulin, antibiotics, and muscle relaxant was initiated. The antibiotics used were as follows: 48.4% of patients received Ceftriaxone, 61.3% received Penicillin V, 12.9% received Amoxicillin/Clavulanic acid, 6.4% received Cefotaxime, and 93.5% received Metronidazole. Sputum cultures were not collected from patients due to the unavailability of this type of investigation in our resource-limited hospital. To control the spasms and to relieve the rigidity, diazepam, a muscle relaxant, was administered to all patients. Baclofen was added in 51.61% of cases to aid in relieving spasms and concomitant pain (Table 3).

Complications of tetanus were documented in 19 patients (61.29%). These include pulmonary complications (pneumonia and respiratory failure) in 12 (38.7%) patients and cardiovascular complications (acute circulatory collapse) in 7 (22.58%) patients. Of 31 patients, 15 (48.38%) were discharged well and 1 (3.22%) was discharged with permanent disability (limb amputation). Fifteen patients died accounting for an overall mortality rate of 48.4%. The overall mean duration of hospitalization was 6 days with a median of 5 days (range from 1 to 26 days). The mean and median duration of hospitalization for nonsurvivors (patients who died) was 2 days (range from 1 to 5 days) (Table 3).

## 4. Discussion

Tetanus is still a major health problem in developing countries including Sudan and leads to high morbidity and mortality. In this study, males were affected more than females (*P* < 0.05); this result is similar to other studies in developing counties [9–12].

Males tend to do many outdoor jobs such as farming and other types of field works; hence, they are more susceptible to injuries. The vaccination of females during child-bearing age may reflect the lower percentage of tetanus among females in our study. Health education on tetanus vaccines is mandatory to raise community awareness. The mean age of the study group was 44 years, indicating that tetanus tends to affect the young age group. Our finding is also related to other studies from the developing countries, which reported similar results [6, 13, 14].

Another important risk factor is the occupation of the patient. Studies from Nigeria and China show the predominance of farmers among tetanus-infected patients [4, 15]. In contrast, this study shows that workers have an incidence of 48.4%, whereas farmers and civil servants have an incidence of 19.4% and 3.2%, respectively.

*C. tetani* enters the body through puncture wounds, lacerations, skin pricks, surgical procedures, or animal bites. In this study, the bacteria enter the body through these contaminated types of wounds in 80.62% of patients. This result is similar to a study from China [4]. The study also delineated that the most common portal of entry was the lower limbs; this explains that *C. tetani* exists in soil and animal feces and thus any contaminated wound in the lower limbs may act as a portal of entry. Our result is similar to other studies [6, 9, 16] and in contrast to [12]. The vasculopathy and ulcerations associated with diabetes may increase the chance of getting the disease; in this study, only 2 patients were diabetic. In addition, hypertension was also reported to be associated with tetanus infection according to Rogers and Frykberg [17].

In this study, the incubation period ranged from 2 to 25 days with a mean of 9 days; this result is comparable with Zielinski and Rudowska, who reported that the incubation period ranged from 3 to 36 days with a mean of 12 days [18]. Hence, the laboratory and imaging studies are of low value; the diagnosis of tetanus should be suspected when the patient presented with a combination of these symptoms/signs: generalized muscle spasms, convulsions, lock jaw, drooling, uncontrolled urination and defecation, arching spasms of the back (opisthotonus), dysphagia, trismus, and abdominal/chest/back pain. In this study, lock jaw was the most common presentation accounting for 54.8%. Feroz AHM and Komolafe MA [11, 13] reported that trismus, dysphagia, and body stiffness/spasm were the most common presentations.

Treatment of tetanus requires an equipped intensive care unit with well-trained medical and nursing staff; unfortunately, this was not accessible in our hospital. In the current study, all patients with an identifiable portal of entry underwent surgical debridement to prevent further elaboration of the toxin. In terms of antibiotics, the most common antibiotics used were Penicillin V and Metronidazole. Metronidazole has been found to slow the disease progression and reduce mortality, whereas Penicillin V potentiates the effect of tetanus toxin by inhibiting the type-A (GABAA) receptor for gamma-amino-n-butyric acid [19, 20]. Diazepam, a drug of the benzodiazepines class, was administered to all patients; it reduces anxiety, relaxes muscles, causes sedation, and prevents cardiopulmonary complications, thus improving the patient outcome [20]. The usual dose was 10 to 30 mg every eight hours. Human tetanus immunoglobulin can neutralize toxins and thus reduce the severity and shorten the course of illness; in this study, HTIG was administered to all patients.

The mortality rate in this study was 48.4%; this finding is fairly consistent with those of other studies [16]. Most of the deaths were attributed to sudden cardiac deaths and respiratory complications. Studies in Bangladesh and the United States reported a lower mortality rate than 48% [11, 16, 21]. We assign the high mortality rate in the current study to the lack of resources. The survival rate in our study was 51.61%. One of them suffered permanent disabilities; he required below-the-knee amputation for right foot sepsis that did not respond to the medical treatment. The overall mean duration of hospitalization was 6 days, which is low compared to other studies [9, 12, 14].

## 5. Conclusion

In Sudan, tetanus remains a disease with high morbidity and mortality. As tetanus is a vaccine-preventable infectious illness, a routine vaccination and appropriate wound care must be brought to attention. The incomplete vaccination status among study participants, along with inadequate management and lack of equipped resources, leads to a devastating outcome.

## Figures and Tables

**Table 1:** Demographic characteristics of the patients.

Parameters of the patients	Frequency	%
*Age*		
Less than 20 years	2	6.5
From 20 to 29 years	4	12.9
From 30 to 39 years	6	19.4
From 40 to 49 years	7	22.6
From 50 to 59 years	5	16.1
From 60 to 69 years	4	12.9
More than 69 years	3	9.7

*Gender*		
Male	23	74.2
Female	8	25.8

*Occupation*		
Free worker	15	48.4
Farmer	6	19.4
Civil servant	1	3.2
Housewife	7	22.6
Student	1	3.2
Retired	1	3.2

**Table 2:** Disease characteristics of the patients.

Parameters of the patients	Frequency	%
*Etiology*		
Wound injury (puncture, prick, or laceration)	20	64.51
Snake bite	3	9.67
Scorpion sting	1	3.22
Local surgical procedure	1	3.22
No identifiable cause	6	19.35

*Site of the injury*		
Lower limbs	15	48.38
Upper limbs	8	25.80
Trunk	2	6.45
Unknown	6	19.35

*Comorbidity*		
Diabetes mellitus	2	6.5
Hypertension	1	3.2
No comorbidity	28	90.3
*Common presenting complaints*		
Lock jaw	17	54.8
Muscle spasm	16	51.6
Neck pain and stiffness	14	45.2
Convulsions	12	38.7
Back pain	8	25.8
*Mean length of hospitalization (days)*	6.70	

**Table 3:** Treatment characteristics of patients.

Parameters of the patients	Frequency	%
*Antibiotics*		
Ceftriaxone	15	48.4
Penicillin V	19	61.3
Metronidazole	29	93.5
Amoxicillin/Clavulanic acid	4	12.9
Cefotaxime	2	6.4

*Muscle relaxant*		
Diazepam	31	100
Baclofen	16	51.61
Mechanical ventilation	0	0

*Complications*		
Pneumonia	4	12.90
Respiratory failure	8	25.80
Acute circulatory collapse	7	22.58
None	12	38.70
Death	15	48.4

## Data Availability

The data used to support the findings of this study are available from the corresponding author upon request.
